# Application and Effect of Virtual Reality Technology in Motor Skill Intervention for Individuals with Developmental Disabilities: A Systematic Review

**DOI:** 10.3390/ijerph20054619

**Published:** 2023-03-06

**Authors:** Wangqian Fu, Chenying Ji

**Affiliations:** 1 School of Special Education, Faculty of Education, Beijing Normal University, Beijing 100875, China; 2 School of Education, Beijing Sport University, Beijing 100084, China

**Keywords:** virtual reality technology, obstacles to development, motor skills, intervention

## Abstract

Children and adolescents with developmental disabilities are often impaired with motor function, and motor skills are related to the performance of daily living activities, and their defects may limit social participation and reduce the quality of life. With the progress of information technology, virtual reality is used as an emerging and alternative intervention method for the intervention of its motor skills. However, the application of this field is still limited in our country, so it is of great significance to systematically analyze the foreign intervention in this field. The research used Web of Science, EBSCO, PubMed, and other databases to search the literature published in the past ten years on the application of virtual reality technology in the motor skill intervention of people with developmental disabilities, and analyzed the demographic characteristics, intervention target behavior, intervention time, intervention effect, and statistical methods. The advantages and disadvantages of research in this field are summarized, and on this basis, the reflection and prospects for follow-up intervention research are put forward.

## 1. Introduction

Developmental disability refers to a group of disorders with impairment in the physical, learning, language, or behavioral domains including ADHD, autism, cerebral palsy, intellectual disability, and other mental development disorders and they have challenges in their daily lives, motor function, and learning function [1]. It is vital to utilize appropriate intervention to foster the development of relevant skills in people with developmental disabilities in order to help them develop more thoroughly and better adapt to society. Motor function impairment is frequently present in people with developmental impairments [2]. As performance in daily living activities is correlated with motor function, these abnormalities may restrict social involvement and a lower quality of life [3]. Motor skill refers to the general term of the process of individuals or groups engaging in various physical activities, that is, the general term of the process of integrating or regulating the resources of motor behavior according to the rules of skills including the four basic elements of goal, perception, action, and practice [4]. Running, jumping, throwing, pulling, pushing, and other basic skills are the basic structure of motor skills. The principles of exercise rehabilitation include repeated group exercises, exercise dosage, task-oriented and goal-specific functional training, random variable exercises, multisensory stimulation, and increasing difficulty [5]. Traditional exercise rehabilitation is resource-intensive and requires a long duration and high repetition of training [6]. However, for people with developmental disabilities, sustained participation in repetitive tasks is challenging [7].

With the progress of information technology, virtual reality (VR) is an emerging and alternative intervention means [8]. VR refers to the technology of using one system to imitate another real system, which can provide a wide range of activities and action scenes [9]. Through the establishment of artificially constructed three-dimensional virtual environment, users interact and influence each other with objects in the virtual environment in a natural way, greatly expanding the ability of human beings to understand, simulate, and adapt to the world [9]. The research by Bryant et al. showed that VR is an attractive way to provide immersive and interactive training that can provide functional skills for the real world [10]. In recent years, VR technology has been gradually used in the intervention of developing motor skills for people with disabilities and has played a key role. Several studies have shown that VR technology has a positive effect on motor intervention for children with developmental disabilities [11,12]. VR-based motor rehabilitation can provide repetitive high-intensity tasks and immediate sensory-motor feedback on the children’s activity performance, which is an important part of children’s learning. In addition, the use of VR games can stimulate the children’s motivation to learn, improve attention, and adjust the task difficulty according to the user in a pleasant and fun environment to increase the attractiveness of the intervention and keep them actively engaged in the activity. Due to the huge growth of immersive technology, the Coie research report on virtual reality technology suggests that VR and other mixed reality will be as widely used as mobile devices by 2025 [13]. Although there have been some review studies documenting the positive results on individuals with the physical health of developmental disabilities [14,15], their study group was limited to the children with a specific disability. Thus, it would be difficult to generalize their findings to the whole DD group and the validity is also limited. Thus, the effect of the application of VR in motor intervention for people with developmental disabilities is unclear. As an extension to these two aforementioned reviews, the present study therefore aimed to systematically summarize VR-based interventions on the motor skills of individuals with developmental disabilities, which can enlighten the future applications in this field.

## 2. Method

The systematic review was performed following the Preferred Reporting Items for Systematic Review and Meta-Analysis Protocols (PRISMA-P) 2015 statement [16].

### 2.1. Search Strategy and Eligibility Criteria

The following terms and phrases (“developmental disability” OR “autism” OR “intellectual disability” OR “cerebral palsy” OR “developmental coordination disorder” OR “Down’s syndrome”) AND (“Virtual Reality” OR “VR” OR “Virtual environment”) AND (“motor” OR “physical”) were searched in the Web of Science, PubMed, and EBSCO databases as keywords. The specific standards are as follows. (1) The publication period was from 2010 to December 2021. Although considering the rapid development of VR, we found that the other systematic review still contained the studies from inception up to the time for research [14,15]. A systematic review of the effects of motor skill intervention on the basic motor skills of children and adolescents with intellectual disabilities searched the studies on this topic, and the selected articles were published since 2012 [17]. Based on this, we limited the publication time for studies from 2010 to 2021. (2) They were intervention studies on people with developmental disabilities. (3) The articles used VR-integrated exercise equipment. (4) Clear intervention purpose, intervention object, intervention process and intervention results were reported.

### 2.2. Data Extraction

The screening process was independently conducted by two reviewers. We screened the titles of the potential relevant articles and then further screened the abstracts of these articles. Duplicate articles were removed. To narrow down the list of potentially pertinent papers, full-texts were consulted. The authors, year of publication, participant characteristics (sample size, gender, mean age/age range, types of DD, level of DD), interventions (kind of VR, time, frequency, duration, intensity), outcomes, and related measures were all included in the data that were extracted.

### 2.3. Study Risk-of-Bias Assessment

The process of assessing the risk of bias for each study was independently performed by two reviewers using eight quality assessment tools from the previous literature (Table 1) [17,18,19,20,21,22,23,24,25,26,27,28,29]. When the project was clearly described and presented, the study was recorded as “1” (positive), and if the project description was inadequate or missing, the study was recorded as “0” (negative). Among all of the eight indicators, randomization, pre-test/post-test study designs, study retention, and the use power analysis were considered as the most important factors as they had the most profound impact on the research results. The final score for each study was calculated from the sum of all “1” evaluations. The studies that were evaluated as ”high quality and low risk of bias” were signified by a score greater than the median score of 5, whereas “low quality and high risk for bias” studies were those that scored lower than the median score of 5.

Finally, 13 articles were obtained. Based on these studies, the relevant analysis was carried out in order to promote the relevant research and technology (Table 2).

## 3. Results

### 3.1. Study Selection

**Figure 1:**
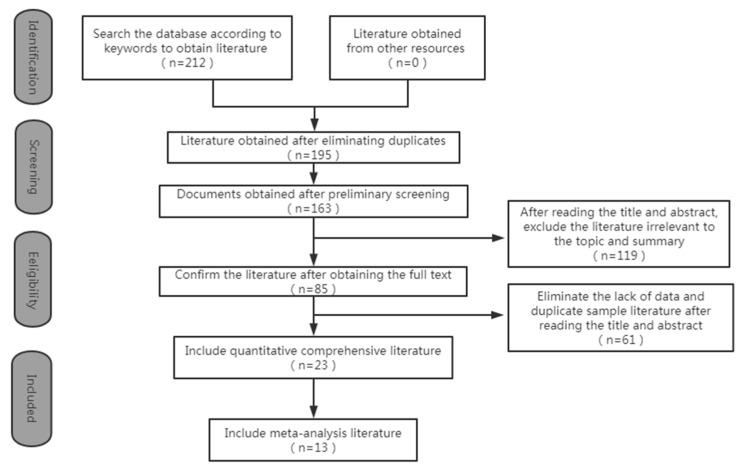
Flowchart of the selection process.

By meeting all of the predetermined eligibility criteria, 13 articles met the inclusion requirements and were included in this review (Figure 1).

### 3.2. Demographic Characteristics

A total of 480 people with developmental disabilities were tested including 191 people with cerebral palsy or brain injury, 114 people with autism, 84 people with intellectual disabilities, and 46 people with other developmental disabilities. The remaining number was the type of disability not reported. Among the included documents, the population with developmental disabilities under 18 years of age accounted for 61.54% of the total, the population with developmental disabilities above 18 years of age accounted for 30.77% of the total, and the population over 18 years of age accounted for 0.76%.

### 3.3. Quality and Risk of Bias Assessment

The risk assessment table for bias among the included studies is shown in Table 1. In detail, the study quality ranged from three points to seven points with a mode of five points. Nine of the included studies scored equal to or greater than the median score of five and were therefore considered high quality, while four of the included studies scored lower than the median score of five and were consequently considered low quality. However, only seven articles accounted for the analysis of missing values, six articles employed a power analysis prior to experiment, and there were not many studies on follow-up of 6 months after intervention. The low scores were attributed to missing data, the absence of a power analysis, and a lack of follow-up [31].

### 3.4. Characteristics of Intervention

Further analysis of the intervention method, target behavior of intervention, time, cycle, and frequency of intervention showed the following results:

#### 3.4.1. Target Behavior of Intervention

A total of 33.3% of the interventions were to improve the functional balance and activity ability of people with developmental disabilities and to improve the motor function of a certain limb of children with brain injury; 20% of interventions were designed to predict the benefits of motor control and increase the physical activity duration in people with developmental disabilities. There are also studies to improve motor proficiency, visual integration, and sensory integration. The overall goal of the selected literature interventions was to improve the level of motor skills.

#### 3.4.2. Intervention Methods

Most of the intervention studies are human–computer interaction by wearing head-mounted sensors or holding handles. According to the program set on the computer in advance, researchers have used 3D computer graphics technology to simulate the tactile feeling by using tactile devices [26]. The researchers relied on the virtual reality system on the computer to simulate the real situation in the form of games to make the participants have a stronger sense of experience. The subjects completed the procedural tasks, that is, carried out the corresponding physical activities, and finally improved the sense of satisfaction and achievement through the reinforcement, in order to promote the development of motor skills. During the intervention, personalized scenarios are usually set up, and therapists tailor personalized language guidance according to the situation of the participants.

For example, in the experiment of intervention on the upper limb motor function of children with cerebral palsy, 10 children in the intervention group received virtual reality rehabilitation therapy, the RAPAEL Smart Kids program, video games combined with traditional occupational therapy (COT) for rehabilitation therapy, while seven children in the control group only received COT alone. VR-based rehabilitation combined with COT covers a series of sports [24], among which RAPAEL Smart Kids is a sensor-based rehabilitation training tool suitable for children with nervous system diseases. This wearable SG can sense wrist movements including forearm pronation/supination, wrist flexion and extension, and wrist radial/ulnar offset. In addition, RAPAEL Smart Kids can be paired with 35 video games (such as fishing, sorting, driving, cooking, cutting, cleaning, painting, playing an instrument, puzzle matching, etc.) to induce targeted movements of the paralyzed wrist and forearm. The difficulty of the game may not be suitable for some special children, and may cause them to lose patience. Each game needs to be classified according to cognition, activity, coordination, and time. According to the judgment of the occupational therapist, the difficulty level and treatment time of each game should be adjusted. The game provides real-time feedback and induces players to generate excitement, which can maintain the learning motivation and motivation of the subjects to continue to improve their physical and skeletal health after the intervention.

In another intervention for children with cerebral palsy, the intervention group was also compared with the experimental group. The intervention group needed a strap wrist attachment and two inertial measurement unit sensors on the back of the hand and the distal forearm, along with the associated software. When the subject moved the upper limb while wearing the device, the avatar would follow the real arm movements on the screen. At the beginning of training, upper limb ability was assessed using a virtual reality device to determine the initial difficulty level. After that, the difficulty level of the training scene was adjusted according to the performance parameters of each individual in each training stage. Both auditory and visual feedback were provided on a computer screen during and after the practice. During each session with the virtual reality system, the therapist helps the child put on the device, motivates them, and prevents them from using the opposite limb during training. In the intervention using virtual reality exercise games to increase physical activity duration in high school students with IDD, the VR exercise game platforms used by the researchers were the Virzoom exercise bike and HTC VIVE VR Google Glass [29]. The Virzoom platform allows people to use purpose-built stationary bikes as controllers for a variety of VR games. For example, the faster you pedal on a stationary bike, the faster the vehicles in the game (racing cars, horses, helicopters, bicycles) will go. When users pedal the bike, they can use the VR headset to obtain a 360-degree view of the interactive game world around them. The researchers collected data on the participants’ heart rate changes and energy expenditure, and compared the baseline and intervention levels for visual analysis [29].

In the intervention experiment to explore the influence of virtual reality technology on children with Down syndrome, a wireless controller was used to interact with players through a motion detection system and an avatar in a video. An acceleration sensor embedded in the controller responds to changes in speed, direction, and acceleration/deceleration, enabling participants to interact with the game while performing different actions. The actions made by children can be captured and reproduced on the screen using infrared light sensors mounted on the top of the TV. The feedback provided by the TV screen as well as the opportunity to observe their own movement in real-time generated positive reinforcement, which facilitated training and task improvement [22]. Pre-training involves obstacle crawling and using different body positions (kneeling, half-kneeling, and standing) to throw the ball. Walking activities include forward, backward and side walking, online walking, animal walking (such as monkeys and crabs), footstep and galloping, strengthening anti-gravity muscles through skateboard games, sit-up exercises and moving pins in different ways, fine motor activities include imitating designs, cutting with scissors and other activities.

In the experiment of the virtual reality training intervention to predict motor control in children with developmental coordination disorder, the data collected in the pretest included the completion results of the hand rotation task (faster reaction time) and sword task (better final effect). After that, the intervention group was given the virtual reality intervention for two months, and then the post-test and two-month follow-up test were performed as before the pretest. Among them, the hand rotation task refers to the stimuli of randomly presenting hand images (9 × 8 cm) in six different directions and different views (back and palm). Participants placed their hands on two buttons (left arrow and right arrow of the keyboard), pressed the corresponding buttons by detecting whether the presented stimulus was left or right, and measured the reaction time to measure the results. The sword task involves taking a wooden sword from a table and inserting it into a hole in a wooden box with a sheet of paper in front of the box with a diagram of the six directions in which to place the sword. The emphasis is on the effect of the angle of rotation of the sword on the choice of grip, with the number of times ending in a comfortable position being the measure of outcome [23].

#### 3.4.3. Time, Period and Frequency of Intervention

In accordance with the standard of 4 weeks in January, the intervention cycle in all documents was converted into weeks. The above documents were analyzed: 7.69% of the intervention less than a week, 15.38% of the intervention cycle was 1–8 weeks, and the intervention cycle was greater than 20 weeks greater than 20 weeks. It accounted for 30.77%, and the other seven studies did not report intervention cycles. It shows that greater than 20 weeks is a common cycle of intervention in development obstacles.

Statistics found that one document did not clearly label intervention time, accounting for 7.69%; 38.46% of the literature real intervention time was 20–30 min; 15.38% of the intervention was 60 min, the shortest display intervention time was 4 min, the longest was 90 min. It showed that the design of the intervention duration varied greatly, in which the intervention duration of one and a half hours was divided into two 45-min periods, with a 30-min rest period in between, during which the participants could choose to sit quietly. The eight-minute intervention applied non-immersive virtual reality technology, asking the participants to perform upper body movements in front of a computer camera and interact with the task. The goal was to intercept spheres falling on four rows to the rhythm of a pre-selected song over an eight-minute period.

The frequency of the intervention experiments was varied, with 12.5% of the interventions being intensive as they were conducted daily for a short period. Experiments with interventions on two days per week accounted for 18.8 percent. Additional studies did not report intervention frequency.

#### 3.4.4. The Effect of Intervention

In the experiment that intervened the people with developmental disabilities, the overall effective intervention rate was 87.5%. The effectiveness of the intervention in the cerebral palsy group was not 100 percent. Although the cerebral palsy group performed better on the real task, it performed worse compared to the typically developing peer group. Most studies reported that the intervention of virtual reality sports games increased the time and intensity of the students’ exercise, and effectively improved the motor skills of the disabled group by providing high-intensity, interactive and repetitive exercise training, and significantly improved the sports performance and enhanced the physical health. Repeated evaluation after the intervention showed that under high-intensity and short-term VR intervention, the functional balance and mobility of adolescents with cerebral palsy were improved, and the changes remained unchanged one month after the training [18], that is, the motor skills were maintained.

#### 3.4.5. Statistical Methods

Intervention studies usually explore the effect of intervention through the comparison of data between different groups and before and after the test. The research data statistics combined with ANOVA were the most, accounting for 38.46%. Second, the research using the *t*-test, or the combination of the *t*-test, chi-square test, and U-test, the visual analysis using the baseline and intervention level and the statistical method using standard deviation were 15.38%. In addition, the statistical methods used in the two studies were not clear.

## 4. Discussion

This study searched the foreign research literature on virtual reality technology in the motor skill intervention of children with developmental disabilities, and analyzed the application method, target behavior, intervention time, frequency and period, intervention effect, and statistical method of virtual reality technology in the motor skill intervention of children with developmental disabilities. The purpose is to provide some reference for our country to use this new technology in the field of intervention for children with developmental disabilities in the future.

### 4.1. Demographic Characteristics

VR, as an intervention for motor skills in a group with developmental disabilities, is still relatively new, and although research is rapidly evolving, its benefits and limitations have not been widely studied [32]. The application of VR on the intervention of the motor skills for individuals with developmental disabilities has mainly focused on individuals with cerebral palsy and autism. It may be closely related with the core deficit of people with cerebral palsy or autism. Cerebral palsy is a term for a group of motor development disorders due to a primary brain lesion, which leads to musculoskeletal problems and limitations regarding activities of daily living [33]. The deficit on the motor muscular movement limits them in attending traditional motor training. For people with autism, they have advantages in the visual processing of information and prefer non-vital features [34], showing a strong affinity for technology [35], which makes VR a promising tool for an intervention. Furthermore, more than half of the literature intervention objects were adolescents under 18 years old, indicating that the current relevant research expects to seize the critical period of adolescent motor skill development to achieve better intervention effects.

### 4.2. Characteristics of Intervention

In terms of intervention methods, the above studies all adopted virtual simulation technology intervention methods to improve motor skills in games. A rich environment can promote neuronal plasticity and functional recovery after traumatic brain injury [36], but the methods used in different intervention experiments are different. One study used upper limb movement in front of a computer camera and interacted with the task [19]. In the process of completing the game, it needs to rely on the cooperation of visual motor skills, attention ability, and body movement. There has also been a comparison between the method of combining video games and traditional offline intervention and the method of only traditional offline intervention [24]. In addition, some experiments need to cooperate with interveners during intervention, that is, researchers need to provide personalized guidance [28]. In terms of the use of external devices, some will use handheld game controllers while others will need to wear multi-inertial sensor helmets. Multi-sensory feedback is provided through virtual images, and patients can adjust themselves when performing different tasks, which is conducive to the comprehensive detection of the basic body movement and motor skills of the subjects.

For the cerebral palsy group, the studies have mainly focused on the improvement in gross muscle motor function, balance and mobility, motor work ability, hand function, and forearm bone health [18], mainly focusing on the upper limb motor function. The main reason is that VR technology currently carries portable glasses, helmets, and handles, which mostly rely on the upper limb to operate and are less restricted by the range of spatial activities. However, VR equipment needs few motor functions that can be applied to legs and other lower limbs. For the intellectually disabled group, most of the selected literature intervened in the aspects of motor proficiency, visual comprehensive ability, and sensory comprehensive function [22].

As for the time, period, and frequency of the intervention, the time, period, and frequency of the intervention were related to the age of the subjects to a certain extent. The personalized reinforcement program gradually increased the frequency, amplitude, and speed of balancing needs. Repetition has been shown to be an important aspect of the task, critical to improving performance, and intensive training over several days is relatively more effective.

The literature results showed that through skill transfer and integration into real life situations, the subjects’ motor learning improved, and most subjects after the intervention had significantly better data results on the sensorimotor measures than the control group. Subjects can convey their inner state with the least abstract language by using the visual scale. This is especially important for patients with developmental disorders, because it is sometimes difficult for them to describe their inner world. The challenges of VR scenes can be controlled, and the subjects can control the speed of the game [35]. In an intervention experiment in which a virtual reality program improved functional balance and mobility in adolescents with cerebral palsy, the data showed that functional balance and mobility in adolescents with CP aged 13 to 18 years could be improved by an intensive, short-term VR intervention. The improvement in outcome indicators remained unchanged for at least 1 month after the VR intervention [18]. In motor intervention for children with Down syndrome, the results also showed that VR improved the children’s motor ability, visual integration ability, and sensory integration function. In the intervention of children with developmental coordination disorder, it was also shown that the performance of the VR group was significantly improved compared with the control group through post-test, and these effects were maintained in the subsequent follow-up stage [23]. These results demonstrate the effectiveness of the VR intervention on the development of motor skills in patients.

In the intervention using virtual reality technology, the role of intervening is also particularly emphasized, which plays the role of encouragers, educators, and guides, and plays an extraordinary role in high-quality intervention [37]. The role of the intervener is very important, which directly affects the effectiveness and quality of the intervention. Through VR interaction, the therapist and the patient will experience this scene at the same time, so they can have a common reference point in the treatment work, and accurately grasp the real-time situation of patients with developmental disorders without a lot of language communication [38]. At the same time, the intervener needs to get the subject to take the initiative to exert some control over the activity choice by encouraging the subject to take the initiative to propose and develop the idea and plan of the activity.

## 5. Reflections of VR Intervention

Despite the proven effectiveness of virtual reality intervention, there are still some challenges in its application. First, it is difficult to control the practice time of the participants at home. Most of the subjects in the above studies received the intervention at the study site, but the intensity and frequency of family practice were different from the skills taught in the treatment course. To ensure the effect of the intervention for a long time, the intervention needs to be carried out in more time and places. This can be carried out by having parents record the type and frequency of the physical activity performed in a daily log. Second, there are high requirements for the ability of the intervene-self. The intervener needs to help the person with the disorder understand what is happening in the current situation and guide them to respond appropriately. The task of the intervener should be planned and prepared before the intervention, and the arrangement of the role of the facilitator should be incorporated into the whole training design to improve the treatment effect [39]. Third, there is a lack of long-term follow-up studies. In terms of exercise capacity, it is necessary to maintain regular exercise. Although the immediate effect was obvious under the intensive intervention in a short period of time, most of the interventions have not been followed-up and observed in the later stage, which makes it difficult to ensure the durability of the intervention effect. Therefore, it is necessary for researchers to follow-up on the intervention. Fourth, subjects may experience uncomfortable feelings during the intervention. The most realistic three-dimensional space games will be presented from a first-person perspective, and the participants will feel the visual conflict between a static body and fast movement, and may feel uncomfortable in the virtual reality intervention such as dizziness, disorientation, or being scared by the virtual reality environment [40]. Therefore, it is necessary to further develop virtual reality technology, design more effective auxiliary applications, improve the human adaptability of the game experience, conform to ergonomics, reduce the frequency of discomfort such as “vertigo” and so on, so that users can be intervened in a more comfortable environment.

Novel interventions using virtual reality technology are increasingly being used to optimize exercise rehabilitation through task-oriented practices. Virtual environments provide unique multidimensional media involving multimodal sensory processes in which individuals interact with virtual scenes that correspond to real life situations. The task difficulty level of the experiment can be adjusted to provide an optimal, motivational challenge. Despite some limitations, virtual reality is a viable and potentially effective intervention. At present, virtual reality technology has been applied in various fields of education and teaching, but the application of virtual reality technology in the education and teaching of special children is still limited, and the relevant research and intervention results are relatively few in number. In the future, more attempts should be made to combine virtual reality technology with other intervention methods to focus on the application of virtual reality technology for different types of special needs students. Further research is needed to observe whether a longer intervention period will show greater changes, to extend the training effect to functional tasks requiring similar motor skills, to optimize the factors in the intervention experiment, and to explore the application of virtual reality technology in different types of special needs students. This will help special needs children to improve their adaptability to better participate in social life.

## 6. Limitations

The study had the following limitations. First, all of the included studies were intervention studies, but the approaches of the application of VR varied, which reduced the final conclusion of the effect of VR. Second, the small number of studies included in the analysis limited the examination of potential moderating variables such as cultural region, scales, and virtual reality devices. Third, most of the included studies were single short-term interventions, and there were insufficient long-term follow-up studies, which made it impossible for researchers to evaluate whether the impact of virtual reality on the individuals’ motor skills was stable in the long-term.

## 7. Conclusions

Literature evidence through systematic analysis shows that virtual reality training has a positive effect on motor intervention for developmentally disabled groups. The rapid development of virtual reality technology provides great potential for the development of sensorimotor training strategies in rehabilitation, novel and widely used 3D technology, clinical applicability using simple graphics and real-time feedback, and multimodal sensory feedback provided by virtual images can allow users to make adjustments while performing different tasks and self-observation [22]. The intervention based on virtual simulation technology has been widely used, which allows the intervener to obtain the information of how the subjects react, explain, and interact with the real world in real-time during the intervention process, thus greatly expanding the existing intervention methods and improving the quality and effect of the intervention. The application of VR technology for intervention training steps were structured, progressive, goal-directed, and interrelated. For example, during the intervention, once the participants initially learn to maintain balance on the virtual sports items, more challenging dynamic tasks such as jumping, hitting, and catching can be introduced in time. After mastering the tasks, the participants can better use body feedback to understand the sports results, predict upcoming events, and plan alternative strategies.

Starting, maintaining, and advancing an exercise program can be difficult for most people, and the use of virtual reality in exercise can alleviate several potential barriers. The interactive game function provides fascinating interactive activities, challenges and encouragement, and increases the physical activity of the subjects [41].

The user experience of the subjects was largely positive. No one asked to quit halfway through, and all subjects completed all tasks. There were great differences between individuals in the group of developmental disabilities, and each person had different needs for the development of motor skills. Virtual reality technology can set up motor tasks, motor difficulty, and reinforcement mechanisms that meet the needs of the users according to their individual requirements, which is helpful to improve the intervention effect.

## Figures and Tables

**Table 1:** Design quality analysis.

Articles	Randomization	Control	Pre–Post	Retention	Mission Data	Power Analysis	Validity Measure	Follow-Up	Score	Effectiveness
Brien and Sveistrup [18]	0	1	1	1	1	1	1	0	6	YES
Martins et al. [19]	0	1	1	0	1	1	0	1	5	YES
Hong et al. [20]	1	1	1	0	0	1	0	1	5	YES
Burke et al. [21]	0	1	1	1	0	0	1	0	4	NA
Wuang et al. [22]	1	1	1	0	1	0	1	1	6	YES
EbrahimiSani et al. [23]	1	1	1	1	1	1	1	0	7	YES
Chang et al. [24]	1	1	1	0	0	0	1	1	5	YES
Schmidt et al. [25]	0	1	0	0	1	0	0	1	3	NA
Chebbi et al. [26]	0	1	1	0	0	0	1	0	3	NA
Golomb et al. [27]	0	1	1	0	1	1	1	0	5	YES
Maskey et al. [28]	1	1	1	0	0	1	1	1	6	YES
McMahon et al. [29]	0	1	1	0	0	0	1	1	4	NA
CHOI et al. [30]	1	1	0	0	0	1	1	1	5	YES

1 refers to positive (explicitly described and present in details); 0 refers to negative (inadequately described and absent); YES effectiveness indicates significant positive effect; NA indicates no significant effect; Median score = 5. Retention: retaining more than 70% of the participants; Follow-up: following more than 6 months after experiment.

**Table 2:** List of included literature (n = 13).

Authors (Year)	Country	Sample Size (Gender)	Mean Age/Age Range	Types of DD	Level of DD	VR Platform/Type	Time/Frequency/Duration	Statistical Method
Brien and Sveistrup (2011) [18]	Canada	Total: 4 (NR)	Total: 13–18	3 spastic diplegia and 1 choreoathetosis	NR	GestureTek Health	90 min; 1 times/day; 5 consecutive days	Compare the baseline between the two groups
Martins et al. (2019) [19]	Brazil	Total: 20 (NR)	Total: 6–9	EG: CP CG: TD	NR	MoveHero	8 min; 5 times/day; NR	ANVOA
Hong et al. (2021) [20]	The Republic of Korea	Total: 21 (NR)	Total: 18–50	ID	NR	Looxid Labs	NR	Chi-square test and *t*-test
Burke et al. (2020) [21]	U.S.A	Total: 153 (NR)	AA: 21.71	99 autism/62 intellectual disability/25 ADHD/1 Down syndrome/1 Prader-Willi syndrome /37 NR	The majority of the participants had one disability (70.59%), 1.93% reported no disability, and other participants had multiple disabilities.	ViTA DMF	4 times; NR; 22 weeks	SD
Wuang et al. (2010) [22]	Taiwan, China	Total: 105 (NR) CG: 53 EG: 52	Total: 7–12	DS	NR	Wii sports	60 min; 2 times/week; 24 weeks	MANOVA
EbrahimiSani et al. (2020) [23]	Iran	Total: 40 (40 F) CG: 20 EG: 20	Total: 7–10	DCD	NR	Xbox Kinect 360	30 min; 2 times/week; 8 weeks	ANOVA and MANOVA
Chang et al. (2020) [24]	The Republic of Korea	Total: 17 CG: 10 EG: 7	Total: 7–18	CP	NR	RAPAEL Smart Kids	20 min; 2 times/week; 8 weeks	Analysis of covariance
Schmidt et al. (2019) [25]	U.S.A	Total: 5 (5 M)	Total: 24–34	ASD	More than two kinds of obstacles	Virtuoso SVVR	20 min; NR; NR	Descriptive analysis and interview coding
Chebbi et al. (2014) [26]	Britain	Total:21 (16 F/6 M) CG: 11 EG: 10 (LD)	AA: 23	LD	NR	Sensables Technologies	15 min; NR; NR	ANOVA
Golomb et al. (2011) [27]	U.S.A	Total: 1 (1 M)	15	CP	Severe	5 DT 5 Ultra Glove	20 min; 2.6 times/week; 56 weeks	NR
Maskey et al. (2014) [28]	Britain	Total: 9	Total: 7–13	ASD	Mild	VRE	20–30 min; 4 times; NR	*t*-test
McMahon et al. (2019) [29]	U.S.A	Total: 4 (1 F/3 M)	Total: 14–21	1 Fetal alcohol syndrome/1 ASD/1 DS/1 ID	Mild to moderate	Virzoom and HTC VIVE VR	30 min; NR; NR	NR
Choi et al. (2020) [30]	The Republic of Korea	Total: 80 (39 F/41 M) CG: 40 EG: 40	Total: 3–16	NR	NR	NR	60 min; 1 time/week; 20 weeks	*t*-test

CG, control group; EG, experimental group; F, female; M, man; AA: average age; ADHD: attention deficit hyperactivity disorder; DS: Down syndrome; DCD: developmental coordination disorder; LD: learning disability; CP: cerebral palsy; ID: intellectual disability; ANVOA: single factor analysis of variance; SD: standard deviation; MANOVA: multivariate analysis of variance; RCI: reliable change indicator.

## Data Availability

No new data were created.
